# Overlapping between IgG4-RD and Behçet’s Disease

**DOI:** 10.31138/mjr.31.1.92

**Published:** 2020-03-31

**Authors:** Silvia Suárez-Díaz, Luis Caminal-Montero

**Affiliations:** 1 Internal Medicine Department, Hospital Universitario Central de Asturias, Oviedo, Spain,; 2 Systemic Autoinmune Disease Unit, Internal Medicine Department, Instituto de Investigación Sanitaria del Principado de Asturias (ISPA), Hospital Universitario Central de Asturias, Oviedo, Spain

**Keywords:** IgG4 Related Disease, Behçet’sDisease, Overlap syndrome

## To the editor:

Alazani MB et al.^1^ reported for the first time a case of IgG4-Related disease (IgG4-RD) retroperitoneal mass in a patient with Behçet’s Disease (BD), and they found only one other similar patient with BD, and in that case, an IgG4-RD laryngeal mass.^2^ Similarly, we have previously described another patient with recurrent aphthous stomatitis and IgG4-related laryngitis with the suspected diagnosis of overlapping with BD.^3^

Some conditions that were previously thought to be unique clinical syndrome are now recognized as a clinical manifestation of IgG4-RD. IgG4-RD physiopathological basis is the presentation of autoantigens by plasmablasts or B cells to CD4 cytotoxic T cells, which produce pro-fibrotic cytokines such as IFN-γ, IL1-ß and TGF-ß.^4^ Although BD shares some common features with autoimmune and autoinflammatory diseases, is ultimately caused by disturbance of T-cell homeostasis, especially Th1 and Th17 expansion, as well as Tregs response suppression. Neutrophil activity is increased at the earliest stage of inflammation in the affected organs, as well as the presence of HLA-B*5 and increased IL-17, which appears to play a major role in neutrophil activity.^5^ IgG4-RD is a rare, multiorgan condition characterized by histologic features, and accurate clinical diagnosis can be challenging due to the atypical manifestations or the presence of overlapping autoimmune diseases like BD or antineutrophil cytoplasmic antibody-associated vasculitides.^6^

## References

[1] Alanazi MB Asiri YO Al-Homood IA IgG4-Related Disease coexisting with Behçet’s Disease. Mediterr J Rheumatol 2019;30(4):228–30. [10.31138/mjr.30.4.228] PMC7241657 32467875

[2] Shaib Y Ton E Goldschmeding R Tekstra J IgG4-related disease with atypical laryngeal presentation and Behcet/granulomatous polyangiitis mimicking features. BMJ Case Rep 2013;pii: bcr2013009158. [10.1136/bcr-2013-009158] [PMID: ] [PMCID: ] 23813505 PMC3703034

[3] Suárez-Díaz S Núñez-Batalla F Fernández-García MS Fernández-Llana MB Yllera-Gutiérrez C Caminal-Montero C Aphthous Stomatitis and Laryngitis, Another Form of Presentation of an IgG4-Related Disease? [Article in English, Spanish] Reumatol Clin 2018;pii: S1699-258X(18)30191-8. [10.1016/j.reuma.2018.08.011] [PMID: ] 30297197

[4] Zhang W Stone JH Management of IgG4-related disease. Lancet Rheumatol 2019;1(1):e55–65. [10.1016/S2665-9913(19)30017-7] 38229361

[5] Leccese P Alpsoy E Behçet’s Disease: An Overview of Etiopathogenesis. Front Immunol 2019;10:1067. [10.3389/fimmu.2019.01067] [PMID: ] [PMCID: ] 31134098 PMC6523006

[6] Danlos FX Rossi GM Blockmans D Emmi G Kronbichler A Durupt S Maynard C Antineutrophil cytoplasmic antibody-associated vasculitides and IgG4-related disease: A new overlap syndrome. Autoimmun Rev 2017;16(10):1036–43. [10.1016/j.autrev.2017.07.020] [PMID: ] 28780079

